# Risk of Bias in Experiments, Quasi-Experiments and Natural Experiments Across Disciplines: Discussion Paper and Assessment Framework

**DOI:** 10.1177/18911803261435894

**Published:** 2026-04-18

**Authors:** Hugh Sharma Waddington, David B. Wilson, Terri Pigott, Ariel M. Aloe, Peter Tugwell, Vivian Welch, Gavin Stewart

**Affiliations:** 1 Department of Population Health, Planetary Health Group, 4906 London School of Hygiene and Tropical Medicine, London, UK; 2 Criminology, Law and Society Department, 3298 George Mason University, Fairfax, VA, USA; 3 School of Public Health and the College of Education & Human Development, 1373 Georgia State University, Atlanta, GA, USA; 4 College of Education, 4083 University of Iowa, Iowa City, IA, USA; 5 Department of Epidemiology & Community Medicine, Faculty of Medicine, Bruyère Research Institute, University of Ottawa, Ottawa, ON, Canada; 6 The Campbell Collaboration, Philadelphia, PA, USA; 7 Science for Sustainable Solutions, 5994 Newcastle University, Newcastle Upon Tyne, UK

**Keywords:** risk of bias, randomized controlled trial, quasi-experiment, natural experiment, systematic review, meta-analysis

## Abstract

The evidence we provide to support decision-making should be rigorously appraised so that the findings are shown to be valuable. We discuss the risk of bias in impact evaluations on social and natural science topics – that is, studies using a variety of experimental, quasi-experimental and natural experimental approaches to quantify the causal effect of an intervention, program or policy on an outcome of interest. Existing tools to facilitate evaluation of the risk of bias are usually conceptualized to assess either randomized controlled trials (RCTs) or non-randomized studies of interventions, often called quasi-experimental designs (QEDs) or natural experimental evaluations, but not both. The tools do not adequately reflect how common sources of bias might be addressed in impact evaluations in the social and natural sciences, or the bias sources particular to certain types of design, such as participant reactivity to researcher observation in a trial, or when modelling incorporates known selection mechanisms other than randomization, such as subversion of the assignment rule in a discontinuity design. We present a heuristic to assist reviewers in assessing the confidence in causal inferences. Our approach emphasizes four common sources of bias across RCTs, QEDs and natural experiments – the equivalence of groups, the fidelity of study conditions, the adequacy of measurement, and the reporting of analyses – and we provide signaling questions to evaluate these sources of bias for particular types of study. The approach should be adapted to suit interventions and review topics of interest.

**Highlights**

**What Is Already Known**

A wide range of methods is used to evaluate causal inference in the social and natural sciences, including randomized controlled trials (RCTs), quasi-experimental designs (QEDs) and natural experiments. Existing risk-of-bias tools are usually conceptualized to assess either RCTs or QEDs and other types of evaluation. They do not adequately reflect common sources of bias across study designs, or sources particular to the types of designs commonly used in social and natural science and related fields.

**What Is New**

We discuss assessment of the risk of bias in impact evaluations on social and natural science topics and related fields like economics, education and population health. We provide a heuristic for the evaluation of causal inference that is appropriate for the range of randomized and quasi-experimental designs commonly used in impact evaluation, which incorporates the equivalence of groups, fidelity of study conditions, adequacy of measurement, and reporting of analyses.

**Potential Impact for Policy, Practice and Research**

Two characteristics of impact evaluation research on social and natural sciences and related fields are that treatments are often allocated using group level or “targeted” selection mechanisms, and a multitude of study designs is used to evaluate impact. By discussing how the different categories of bias interact for different types of study design, treatment selection mechanism and causal pathway, we aim to enable evaluation of the risk of bias from a more policy relevant perspective. This is a hitherto neglected pre-requisite for robust synthesis in an interdisciplinary context.

## Introduction

This article is about the validity of the causal inferences provided in impact evaluations using random assignment (randomized controlled trials, RCTs), quasi-experimental designs (QEDs) and natural experimental evaluations.^1^ Assessing their credibility is a central aspect of policy research in the social and natural sciences, health and related fields, helping decision makers measure what difference an intervention, practice, program, or policy made to outcomes – that is, its effectiveness. The bulk of systematic reviews conducted by the Campbell Collaboration and related organizations assess empirical evidence to answer questions about effectiveness. For these types of reviews, the review team is required to evaluate the risk of bias in the causal inferences made in a study (Campbell Collaboration, 2021; Higgins et al., 2021; Littell & White, 2018) through assessment of the design, conduct and reporting of evidence, to ensure that the findings are shown to be valuable for decision making.

The ability of a study to provide the basis for a valid causal inference depends on its design, such as whether treatment was randomly allocated to study participants or other units of analysis, or whether baseline measures were collected, and how well the study was conducted, such as whether the treatment selection mechanism (e.g., assignment by randomization or a threshold on a scale variable) was subverted, or measurement was subject to reporting bias. Thus, a quality systematic review will critically appraise the credibility of the inferences made and do so transparently, through the use of a risk-of-bias assessment tool.

A recent review of systematic reviews of interventions published in the Campbell library found they used a variety of tools to assess the risk of bias (Wang et al., 2021). A large number of risk-of-bias tools is used in related fields like environment (e.g., Boyd et al., 2022), health care (Farrah et al., 2019) and social policy (Waddington et al., 2017). This diversity in the risk-of-bias tools used across reviews reflects the need of review teams to adapt the tool to the potential sources of bias relevant to the specific intervention being studied and the research designs used within that research area.

Systematic reviews of the effectiveness of an intervention, practice, program, or policy are usually required to search for and include RCTs when they are available (Campbell Collaboration, 2021). In medicine, there is broad agreement regarding the methods for assessing RCTs (Higgins et al., 2021). While there is great recognition of the value of RCTs in fields like economics, education, environment and health, a majority of studies eligible for inclusion in a systematic review on these topics might use non-randomized assignment (quasi-experiments, natural experimental evaluations or simply “observational studies”). These types of studies are empirically valuable in informing decisions on social and related topics. However, there is much less agreement regarding the methods for assessing RCTs, quasi-experimental designs and natural experimental evaluations in social and natural sciences and related fields. Compounding the challenge of assessing the risk of bias is that existing risk-of-bias tools tend to focus on either RCTs or non-randomized studies, but not both. However, the possible sources of bias that affect causal claims are applicable regardless of study design. An assessment framework that is applicable to different types of design would be valuable.

We are concerned that the methods established in medicine to assess the risk of bias are inappropriate in the context of interventions outside medicine, particularly where synthesis demands pooling of disparate study designs. Systematic reviewers of intervention effectiveness need an approach to assess study designs and risk of bias for both RCTs and QEDs, which is adaptable to the relevant sources of bias within the research being synthesized. The approach will ideally assess bias categories consistently, and can draw on, and further develop, categories expounded in existing tools. These biases can pervade entire literatures of particular types of design – for example, participant reactivity effects in trials, or lack of group equivalence in study designs with self-selection into treatment. Hence their measurement across designs can also provide evidence to evaluate the consistency of an evidence base. The resulting assessments can be incorporated into strength of evidence assessments such as the Grading of Recommendations, Assessment, Development, and Evaluations (GRADE) (Schünemann et al., 2019).

This paper discusses a framework for assessing the risk of bias in these studies. The objective is to help those involved in reviewing RCTs, quasi-experiments and natural experiments to evaluate bias consistently and appropriately. Our work grew out of a combination of literature review and discussion among experts across fields including criminology, ecology, economics, education, environmental science and health. The next two sections discuss the risk-of-bias approaches used in systematic reviews of interventions published in the Campbell Library, and the key aspects of impact evaluations in social and natural sciences which underpin the framework we have developed for evaluating the risk of bias. The fourth Section presents the framework itself, including signaling questions to enable reviewers to evaluate the likelihood of bias, for randomized and non-randomized, historical and contemporaneous comparison group designs. The fifth Section discusses practical considerations and the possible relationship between the risk of bias and the treatment effect estimate, and the final Section concludes.

## The Application of Risk-Of-Bias Tools on Social and Natural Science Topics

In this section we review the risk-of-bias approaches commonly used in systematic reviews of interventions, including Cochrane’s tools and those published in the Campbell Library. The effect estimates in quantitative causal studies are characterized with reference to an explicit counterfactual. The studies therefore need to be evaluated according to adequacy of the counterfactual, that is, the extent to which the “control [or comparison] group provides an unbiased estimate of the average *potential outcome* that experimental units would have attained had the treatment not been applied to them” (Cook & Steiner, 2010, p. 57) In practice, this means the extent to which the study is able to address, by design or in the methods of analysis, threats to group equivalence, which arise through the processes of assignment and adherence to treatment, and the consequences of differential selection into and out of measurement, known commonly as selection bias (Shadish et al., 2002). It is crucial that a study is designed to account for these sources of bias in estimating the potential outcome in the counterfactual group (Rubin, 1974).

Risk-of-bias tools, such as Cochrane’s tools for RCTs (Sterne et al., 2019) provide decision algorithms for determining the likelihood that a study is biased – that is, that the estimated effect might deviate from the true effect in expectation – overall and for specific bias categories. While multiple tools are used in the assessment of quantitative effectiveness studies, they often specify different categories of bias for RCTs and other types of studies, even though the underlying sources of bias across these studies are usually the same. We group these sources under the equivalence of groups (selection bias), the fidelity of study conditions (performance bias), the adequacy of measurement (information bias) and the reporting of analyses conducted (reporting bias).

Risk-of-bias tools make use of “signaling questions” that enable reviewers to assess the likelihood of bias across a range of design and analysis approaches used in evaluation research. Many tools evaluate QEDs according to their ability to control for observed sources of selection into treatment, ignoring approaches that can address unobserved sources too. For example, Cochrane’s tool for assessing the risk of bias in non-randomized studies of interventions (ROBINS-I) was developed for studies where confounding is addressed through direct control for observed characteristics (e.g., incorporating confounding variables as covariates in the model) (Sterne et al., 2016). It was not designed, and is not sufficiently applicable, to the range of QEDs and natural experiments that can control for some or all unobserved sources of selection bias by design or in analysis (e.g., discontinuity designs, interrupted time-series, difference-in-differences, synthetic control or instrumental variables estimation).

There has been a reappraisal of quasi-experiments and natural experimental evaluations in recent years, motivated by the further development and empirical evaluation of credible estimation methods for non-randomized evaluations, and the recognition of the need to draw on evidence that is relevant for the real world of policy and practice (Craig et al., 2025; Sharma Waddington et al., 2022b; Tugwell et al., 2017). When we started work on this discussion paper, we assessed the risk-of-bias approaches used in Campbell reviews to date, which then formed the basis of the approach we subsequently developed. We found that 80 (81%) of 99 Campbell systematic review reports had included quasi-experiments (Villar & Waddington, 2019). Authors were found to have used different tools to assess the risk of bias for included studies, roughly corresponding to the group coordinating the registration process. Thus, for example, many Crime and Justice Group authors used tools they developed for the specific purposes of the review in question (e.g., Wilson et al., 2016), whereas International Development Group authors largely used the tool developed by that group (Hombrados & Waddington, 2012).

The categories of bias analyzed in the systematic reviews also varied (Table 1).^2^ This variability appears to be due to some tools omitting important categories, rather than inappropriate application of appropriate tools by the systematic reviewers. Baseline equivalence was assessed in 86% of Campbell reviews. In addition, over 80% of reviews assessed biases in outcomes measurement (although fewer than 50% assessed biases in intervention measurement too) and 70% assessed selection of the reported result. Factors relating to equivalence of groups over time, such as losses to follow-up (attrition), were assessed in around 70% of reviews. But differential selection into the study, when all follow-up data are missing for some groups who would otherwise have been eligible for conditions (e.g., in a retrospective study that selects into the study based on the availability of outcome data), was not usually addressed specifically. To take another example, fidelity of study conditions, which may arise due to non-adherence of participants to intended intervention (e.g., participant switches between comparator and treatment), the consequences of exposure to treated individuals or their outcomes (spillover effects), co-interventions received differentially, or fidelity of treatment was assessed in fewer than half (40%) of the reviews. In addition, participant or practitioner reactivity in response to observation was not usually addressed, either in RCTs or QEDs, with the exception of some of the reviews that used Hombrados and Waddington’s (2012) tool.

**Table 1:** Risk of Bias Categories Assessed in Campbell Systematic Reviews (Percent)

Bias category	Crime & justice	Education	International development	Social welfare	All topic areas
Equivalence of groups at baseline	100	71	92	83	86
Equivalence of groups post-baseline (e.g., attrition)	53	86	62	83	71
Fidelity of study conditions	13	48	58	28	40
Measurement of intervention and outcome	27	29	50	72	45
Selection of reported result	33	62	85	83	69

*Note.* Figures are percentages of reviews that assessed the risk-of-bias category by topic area.

Some attempts have been made to design critical appraisal tools for social experiments that are relevant for these designs (e.g., Valentine & Cooper, 2008). Tools have also been created to evaluate RCTs and observational studies consistently in medicine (Faillie et al., 2017). However, our conclusion is that existing risk-of-bias tools are insufficiently nuanced to assess bias in studies of interventions in the social and natural sciences, whether defined as RCTs, QEDs, natural experiments or observational studies.

## Causal Inference in the Social and Natural Sciences and Related Disciplines

A range of methods is used to evaluate biases in health, natural and social science literatures, none of which, in our view, adequately and consistently represents the sources of bias across the variety of designs included in systematic reviews. Before presenting the risk-of-bias evaluation framework itself, we discuss three key aspects of our approach for assessing impact evaluations on social and natural science topics, and how these differ from approaches used in medicine. These relate to the adequate consideration of non-randomized treatment selection mechanisms; evaluating participant and practitioner reactivity in response to research observation; and the role of the theory of change in motivating the specific items included in the bias assessment for the review.

### Treatment Selection Mechanisms

A fundamental feature of social and natural science policies, programs, practices and interventions is that many of them are delivered to groups rather than on an individual basis; and even if they are not, the mechanism of selection of participants to treatment conditions may be known and can therefore be modelled. Understanding treatment selection mechanisms helps in assessing the risk of bias because the information needed to model program placement credibly is less than that needed to model individual self-selection into treatment, a more opaque process.

A key step in the assessment of a given study is to identify the method used to assign study participants to conditions specific to the study context. Understanding why some people receive the program and others do not, that is, understanding the treatment selection mechanism, is critical in formulating strategies to measure the selection process (or approximate it, where unmeasured) (Campbell, 1984; Cook et al., 2008). The strongest study designs for causal inference are those that can address observable and unobservable sources of treatment selection. Some designs account for unobservable sources through perfect knowledge about the method of allocation, the classic example being an RCT through its use of randomized assignment.^3^ This knowledge can also prove useful in modeling the selection process in QEDs and natural experimental evaluations too.

We distinguish “targeted” and “self-selection” into treatment, with examples from our work given in Table 2. Targeted (or top-down) selection may occur through randomization, either by researchers or (more rarely) through a public lottery implemented by administrators. Targeted selection may also be done according to a threshold on a pre-test (or some other baseline measure).

**Table 2:** Types of Treatment Assignment Mechanism With Examples

Mechanism	Description	Examples in crime and justice	Examples in sustainable development
Randomized assignment (targeted selection)	Individuals, groups, or geographic areas are assigned randomly by researchers or through the policy process to treatment and control conditions.	Individuals arrested for a drug related offense are randomly assigned to a drug court or routine criminal justice processing.	A public lottery is held to determine which villages are assigned to receive water supply and sanitation improvements during the current fiscal period, and which are assigned to receive the improvements in a future period.
		Small geographic areas within a city are randomly assigned to two different policing strategies.	
Threshold assignment (targeted selection)	Individuals, groups or geographic areas are assigned to the treatment or comparison condition based on a pre-treatment score on a scale variable	Individuals are assigned to pre-trial release or to remain incarcerated pre-trial based on a risk-assessment score.	Households are allocated to receive a subsidy for latrine installation based on their score on an asset index.
Other types of group- and individual-based assignment (targeted selection)	Group-based: All individuals in a specified category are eligible for treatment such as selected communities, geographical locations, demographic characteristics or socioeconomic factors.	Individuals are assigned to different programs depending on their age, the type of crime, or the jurisdiction where they committed it.	Service delivery points are located in areas where there are greater proportions of people with the most needs.
	Individual-based: Individuals or households are eligible for treatment according to a proxy-means test, selected by practitioners, or by the community.		Households are allocated to receive a subsidy for water installation based on a poverty assessment implemented by community leaders.

Participant self-selection	Among those who are eligible (which may be everyone), individuals decide for themselves whether to participate in the treatment or comparison condition. Certain individuals may be encouraged to participate and participation may include benefits not available to others.	Arrested individuals actively involved in illicit substance use may be provided with a choice from a prosecutor whether to participate in a drug treatment court or to receive traditional criminal justice processing	Water supply is publicly available to all for a small fee.

Source: authors.

Other types of targeted treatment selection mechanisms exist, although they may only be observed imperfectly. For example, treatment selection may be made by program administrators or practitioners using categorical variables which identify target groups using easily identifiable criteria at either the individual or household levels (e.g., sex, age, group membership, criminal history) or at the community level (e.g., specific locations), through proxy means tests, or according to explicit criteria by a third party such as the community leadership or program implementation body (Coady et al., 2004). Some sources of treatment selection can be controlled directly in analysis, assuming they can be measured accurately. For example, information on the individual or group criteria determining assignment (e.g., a threshold, geographical characteristics, socio-demographic or economic factors, criminal history) may be modeled statistically.

Self-selection into treatment occurs from the bottom up, where a program is potentially available for all (or all who satisfy some eligibility criteria) and the decisions to enroll and adhere are voluntary, taken by individuals, households or other groups of participants.^4^ In this case, the selection process is likely to be more difficult to observe or model, if it can be credibly modelled at all. The implication is that a credible impact evaluation will provide some information about how the intervention, practice, program or policy has been assigned, which should also be incorporated into the risk-of-bias assessment.

### Participant Reactivity to Researcher Observation

An important aspect of Cochrane’s tools is whether participants were blind to the condition they received and also whether practitioners (i.e., those providing treatment) and those assessing outcomes were also blind to the condition of any participant with whom they interact. A methodological purpose of blinding is to control for unobservable sources of bias. Each type of blinding is controlling for a different form of bias. Blinding of participants in a medical trial controls for placebo effects and enables the study to isolate the direct effect of the drug from any placebo effect. Blinding of the treatment provider (practitioner) is similar in that it is controlling for the possibility that a provider might inadvertently create expectancy effects for the study participants, altering the outcomes relative to what might happen without treatment. Blinding of the person assessing the outcome, including self-report, is intended to control for any possibility that awareness of a participant’s treatment condition may unintentionally bias outcome assessment.

The need to blind participants to treatment assignment depends on the nature of the treatment and research question of interest. In assessing the efficacy of a drug, it is critical to control for any possible placebo effect – that is, a genuine treatment effect that is not the direct result of a theoretically active element of an intervention (such as the chemical structure of a drug) but because of a change in participants’ expectations. This is because we do not want to administer potentially harmful drugs to individuals when comparable outcomes can be achieved by a placebo. In social and natural sciences, any effect operating on expectations is usually part of the intervention and as such not something to be controlled out of the experiment (independently from whether blinding is possible, although generally in public policy it is not possible to blind participants to condition). An example is police use of body worn cameras. Any effect of these cameras on a police officer’s behavior is presumed to occur through their awareness that they are wearing a camera and that the camera is recording what they are doing. Similarly, any effect of these cameras on citizen behavior is presumed to occur from the citizen’s awareness of an officer wearing a camera which is filming. Thus, it would make little sense to blind police officers or citizens to whether they have been assigned to the body worn camera condition or the control condition (for example, by having police officers wear cameras which are not recording).

This logic extends to most social programs where any expectation effect produced by awareness that the participant is part of the program is also part of its mechanism of effect. That is, the relevant policy question for most evaluations of social and related programs is the total effect of a program relative to either no program or routine practice, including any aspect of this effect produced on participant expectations.^5^ In this context, we would not want to rate a study as at ‘high-risk of bias’ solely based on a failure to blind participants to the conditions. Similar arguments could be made for programs in many other areas, such as cognitive behavioral therapy, selective education, bootcamps in criminal justice, and cash transfer schemes where benefits are tied to children’s continued participation in health care and schooling. A similar argument can be made for treatment providers, such as program staff. So long as any expectancy effects that they may produce are not unique to the evaluation study but would exist in any real-world implementation of the program, then we would not want to control out these effects.

A more insidious problem, which does need to be addressed in evaluations of social and related interventions, is reactivity in response to observation by researchers. The issue is that participants’ and practitioner knowledge that they are part of a research study, or the process of observation used by researchers, could feasibly alter their behavior in a way that affects outcomes or the measurement of outcomes. Study participants might also react differently to performance incentives depending on which group they have been assigned to. These incentives are usually thought to operate favorably in treatment groups (sometimes called “Hawthorne effects”), and they may either incentivize controls through compensatory rivalry (also called “John Henry effects”) or disincentivize them due to resentful demoralization (Bärnighausen et al., 2017). In addition, controls may receive benefits by seeking out treatment (crossovers) or by being exposed to the effects of it (spillovers), or information about it (“testing effects”, as it is the testing that produces the effect, and not the treatment).

Assessments on social and natural science and related topics need methods to evaluate the risk of bias from participants and treatment providers reacting to research observation by altering their behavior and/or reporting, which go beyond simple assessments of whether blinding, a method that might be infeasible (or unnecessary), was conducted.

### The Central Role of the Theory of Change

It should be clear that assessing the risk of bias requires judgment. This judgment is aided when reviewers attempt to understand the theory underlying the causal relationship. We believe risk-of-bias assessment should explicitly involve engagement with the purpose of the systematic review. It can usefully do this by drawing on a method already commonly used, namely the theory of change. Theory of change analysis, a recommended component in Campbell reviews (Campbell Collaboration, 2021), enables reviewers to evaluate the interventions, outcomes and causal processes which relate to the questions posed by the systematic review, which may not necessarily accord with the questions posed by the primary study.

**Figure 1:**
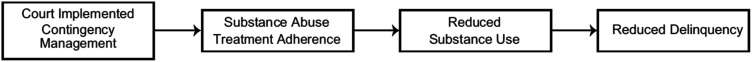
Theory of Change: Change Mechanism for a Juvenile Drug Court. Source: (Wilson, Olaghere, & Kimbrell, 2019)

**Figure 2:**
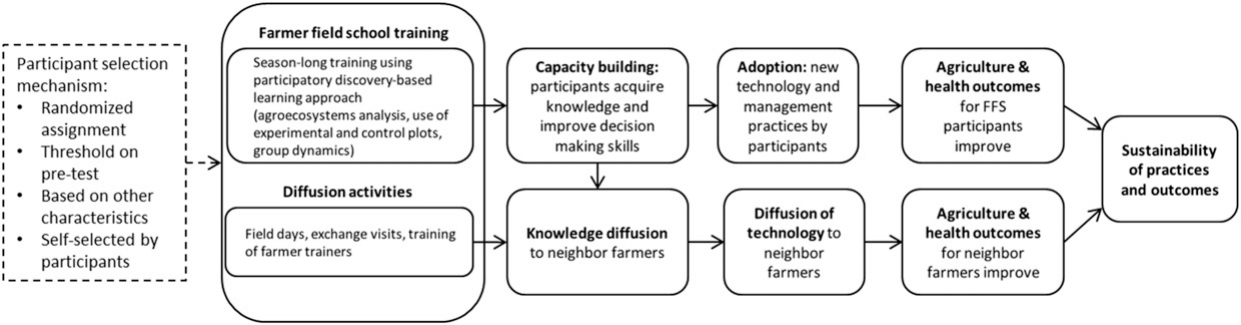
Theory of Change: Field Schools for Improving Farming Practices and Farmer Outcomes. Source: Based on (Waddington et al., 2014)

A theory of change shows how the intervention is anticipated to exert effects on the outcome(s). It is often presented in the form of a logic model (Anderson et al., 2011), which is usually conceptualized to show intermediate outcomes (mediators) along the causal pathway. An example is the causal pathway in Figure 1 for drug courts on juvenile delinquency, which is expected to operate by improving substance abuse treatment adherence and thereby reduced substance use. A theory of change might also incorporate multiple causal pathways for different groups (Figure 2), or assumptions and contextual factors (moderators) thought to influence whether the effect occurs for a particular outcome or its size. This might help in understanding which baseline variables should be modelled to enhance equivalence, or which treatment selection mechanisms have been applied. Some theories of change show intended and unintended effects, as in the example of leakages and substitution in child feeding programs (Kristjansson et al., 2015), or “unpack the intervention black box” more fully than the examples presented here (White, 2018).

In this way, theory of change analysis assists the reviewer in identifying likely outcomes, unintended consequences and contextual moderators of effects at the evidence synthesis stage. This is useful information for risk-of-bias assessments, most obviously in helping identify alternative explanations for changes in the outcome incorporated in estimation, or mediator variables such as measures of treatment uptake or adherence, which should not be incorporated in treatment effect estimation. Unpacking the intervention black box also helps in understanding fidelity of treatment, whether delivered as intended or as would usually be delivered in public programs, but also the effects of observation on participant motivation, such the use of frequent within- and post-intervention research monitoring visits in treatment groups outside of data collection rounds, which may cause biases if these additional visits are not present when the treatment is delivered outside of a research study setting.

Support for the relationship between intervention and outcomes can, in theory, be made through examination of intermediate outcomes or mediator variables. Mediator analysis can be used to help validate or falsify causal claims. Mediation effects consistent with the theory of change help support the credibility of the effectiveness of the intervention, whereas the absence of a relevant mediator relationship undermines the credibility of the treatment effect. An example, relevant for both RCTs and QEDs, is where outcomes are self-reported by participants, and it is not clear whether participants are incentivized to misreport (such as through multiple visits by researchers and disruption of routines). Where outcomes on the causal pathway indicate changes consistent with the outcome of interest, such as observed farming practices in the case of an evaluation of reported agricultural yields, this might provide supportive evidence about the effect measurement.

Understanding the theory of change, including unintended consequences, helps to articulate which outcomes or population groups are unlikely to be affected by the intervention, and therefore the adequacy of falsification methods. An example commonly used is the negative-control outcome function (Lipsitch et al., 2010), which is an outcome that is not thought to be affected by the intervention.^6^ For example, evaluations of the impact of hygiene improvements on diarrhea may also demonstrate no change in the incidence of non-infectious disease. Participant sub-groups can also be used as negative controls, such as measurement among groups for whom outcomes are not expected to evolve in response to the intervention. And where the intervention itself is unpacked as part of the theory of change, it might help identify monitoring that operates differentially between treatment and control (or between ideal and routine settings), thus serving to bias the treatment effect estimate sought.

## Risk-Of-Bias Assessment Framework for Social and Natural Sciences

In this section, we present an approach to assessing the risk of bias for RCTs, QEDs and natural experimental evaluations in the social and natural sciences and related fields. High quality systematic reviews set explicit study design inclusion criteria, and then transparently appraise included studies based on how they are designed, conducted and reported (Campbell Collaboration, 2021). It is worth noting that the sources of bias are relevant across all impact evaluation designs – whether the study used a randomized, quasi-experimental, natural experimental or “observational” evaluation design, was conducted prospectively or retrospectively, using new or existing data, with a contemporaneous or historical comparisons. We therefore break with other published risk-of-bias approaches in two main ways. Firstly, rather than presenting different criteria for RCTs and non-randomized studies, we present an approach in which both types of design are assessed using the same bias categories, reflecting the continuum of study designs laid out in a companion paper (Wilson et al., 2025). Secondly, we suggest questions that reviewers can use to assess important sources of bias across studies as well as questions relating to particular study designs.

We structure this section around eight bias categories, corresponding to four common sources of bias, which affect accurate measurement of the effect of interest (Table A1):

(1) **Equivalence of groups**: incorporating *baseline equivalence*; and *selection into and out of the study*;
(2) **Fidelity of study conditions**: incorporating *participant reactivity*; and *fidelity of the treatment and comparison conditions*;
(3) **Adequacy of measurement**: including *temporal precedence*; and *quality of measurement*; and
(4) **Reporting of analyses**: including *estimation methods*; and *selective reporting.*

We have designed the framework to be consistent with the sources of bias identified previously (e.g., Higgins et al., 2011; Hombrados & Waddington, 2012; Luijendijk et al., 2020; Shadish et al., 2002; Sterne et al., 2016, 2019), corresponding to selection bias (equivalence of groups), performance bias (fidelity of study conditions), information bias (adequacy of measurement) and reporting bias (reporting of analyses). We would usually expect systematic reviews adopting this approach to incorporate signalling questions under each category. However, the criteria on which each of these propositions are verified will depend on the underlying approach for causal inference (study design). Most of the categories refer to complex points, so there are focused signaling questions and sub-questions to articulate each relevant component. The advice to the reader and user is that some questions will matter more for some contexts than others, or there might be additional questions which are important. We are giving examples from our work; what is critical for the reviewer is to adapt the assessment framework to ensure the key questions relevant for the review topic are addressed.

### Equivalence of Groups

#### Baseline Equivalence

In a comparison group design, any non-random differences between the groups at baseline, before the treatment is implemented, may cause bias in the treatment effect estimate. In the Shadish et al. (2002) internal validity framework this is called selection bias. In the directive acyclic graph (DAG) framework this is called confounding (Pearl, 1995).

Only through knowledge of the treatment selection mechanism can one assess the adequacy of methods for addressing potential unobserved differences at baseline. In RCT designs, random assignment to conditions corroborates baseline equivalence, at least probabilistically. However, it is still important to assess the quality of the randomization process. More specifically, assessors of RCTs need information about how random numbers were centrally generated (tossing a coin, drawing from a lottery, using a computer program) and how the allocation was concealed during recruitment of participants to ensure there was no subversion of the randomization process (Eldridge et al., 2016; Higgins et al., 2011). Where a special procedure is used (e.g., blocking [stratification], pairwise matching, unique random draw, multiple random draws etc.) it should be well described and relevant adjustment considered in the analysis (e.g., block [stratum] fixed effects, pairwise matching variables) (Bruhn & McKenzie, 2009). Balance on measured baseline variables between treatment and control groups in an RCT is usually assessed by comparing the mean and standard deviation of these variables. For example, the U.S. What Works Clearinghouse (WWC) standard for baseline equivalence is a difference of 0.25 standard deviations or less (What Works Clearinghouse, 2022). This standard also requires that treatment effect estimates are statistically adjusted for any difference between 0.05 and 0.25 on a baseline variable.

For a discontinuity design, group equivalence is not the primary issue: we know that the groups are different, and we know how they are different, although a balance table of variables measured at baseline is often reported for all subgroups receiving differential treatment at the threshold bandwidth. Rather, adherence to the assignment rule is what is critical as well as a linear relationship (or one that can be linearized) between the selection variable and the outcome. The latter assumption can be verified graphically. Subversion of the assignment rule can also be assessed graphically; for example, bunching of observations at the threshold of the assignment variable is evidence of subversion. For instrumental variables (and quasi-randomized natural experiments), it is important qualitatively to establish that the instrument (or other feasibly “exogenous” identifier) is external – that is, it is only causally related to the outcome by determining selection to treatment. For a one-group design or time-series, the parallel issue is whether the number of pre-test measurement time points is sufficient to establish a credible baseline counterfactual.

For most QEDs, however, establishing baseline equivalence is difficult and often based on untestable assumptions. Some unobserved sources of treatment selection can be accounted for, depending on the nature of the variable and analytic methods used. For example, longitudinal studies using difference-in-differences (DID) models account for unobserved time-invariant differences at the unit of analysis like innate ability, but not any time-varying differences that might confound the relationship between the intervention and outcome, which would be observed by differential pre-test trajectories. Hence, it is standard to undertake assessment of parallel pre-intervention trends in outcomes in DID to establish the credibility of causal inference.

Other known selection rules about how policymakers or program administrators allocated treatment can also increase credibility of the analysis, alongside any baseline or time-invariant characteristics that might be predictive of who received which condition, provided these variables are observed. For example, selection may be modelled in parametric analysis (e.g., multiple regression) or through application of non- and semi-parametric methods (e.g., statistical matching, propensity weighted regression).

Unobservable sources of self-selection, including those factors affecting individual choice to participate in and adhere to treatment, can most effectively be controlled via study design, where factors determining allocation to intervention are precisely known (i.e., RCTs and regression discontinuity design, RDDs). In contrast, attempts to model the self-selection process using observable factors related to both treatment participation and the outcome are likely to be biased. Thus, quasi-experiments where self-selection into treatment and comparison conditions is the primary source of differences across groups, are likely to be at ‘high-risk of bias’ no matter how fancy the statistical methods. Note that it is insufficient to simply include common predictors of the outcome (although these are quite useful). What is critical is modeling any aspect of the selection mechanism that is also related to the outcome.

#### Selection Into and Out of the Study

Any difference between the groups other than the treatment versus comparison distinction at baseline may cause bias. This includes the selection of treatment units or follow-up periods into or out of the study, which causes bias when exclusion of units is correlated with the outcome. Within the DAG framework this is called a collider (Pearl, 1995). Censoring of observations is potentially problematic in studies designed prospectively or retrospectively (after implementation of the intervention). An example is “survivor bias”, where participants who would otherwise have been eligible for the study die or migrate away from treatment and comparison centers. The classic example is from the Second World War, where, following introduction of an improved helmet, more soldiers were observed in hospital wards with brain injuries than before, because the new helmets were saving their lives. A simple analysis would erroneously conclude that the improved helmets were associated with greater injury rates, due to the censoring of the pre-test series of observations by mortality. In economics this is called “endogenous sample selection” (Heckman, 1979). Solutions to this problem include modeling the selection process if data are available on survivors and non-survivors, or simulation if they are not, but often no adjustment is used and the selection process unknown.

The overarching issue when assessing the risk of bias is whether the composition of the treatment and control samples is in any way affected by or related to treatment outcomes. For example, sample selection may also be problematic in multi-level prospective studies (e.g., cluster-RCTs) where participants (“joiners”) are recruited post baseline, after clusters have been randomized to receive treatment, if treatment status is common knowledge for recruiters and/or joiners.

Selection out of the study (commonly called attrition) is potentially problematic in all longitudinal analyses. Addressing these sources of bias satisfactorily usually requires that follow-ups are recorded for all eligible participant units from recruitment onwards (i.e., prior to treatment) together with the reasons given for additions to or drop-outs from the sample. This is most transparently reported using a Consolidated Standards of Reporting Trials (CONSORT) participant flow diagram (Bose, 2010; Hopewell et al., 2025; Moher, 1998) or reporting sufficient information to reconstruct one. Other approaches used to assess whether the losses are random include statistical tests of the relationship between baseline covariates and attrition status, possibly incorporating an interaction term with the treatment to evaluate differential attrition. These approaches assume that observables associated with sample selection are correlated with unobservable factors. There are some thresholds commonly applied to determine whether total and differential attrition is worrisome (e.g., <20% overall attrition, <10 percentage points in difference across groups). WWC also provides a table of cautious and optimistic differential attrition standards given a value for overall attrition (What Works Clearinghouse, 2022).

### Fidelity of Study Conditions

#### Participant Reactivity

We distinguish motivation biases which affect participant behaviors (behavioral bias) from biases in measurement which affect participant reporting, the latter of which are discussed under quality of measurement below. Treatment effects can be biased by research participants’ reactions to being part of a research study. When participants are aware they are part of an impact evaluation, they may alter their behavior in unpredictable ways. For example, this awareness may increase their motivation to perform. This category of biases includes compensatory rivalry on the part of the comparison group, demoralization on part of the comparison group, or improved performance among the treatment group simply because they know they are being studied. Motivation bias is problematic in prospective studies, whether RCTs or QEDs, where the same participants are observed repeatedly and their usual routines disturbed. These effects are less likely to be maintained if the intervention is implemented outside of a research environment. “Testing effects” (also called “survey effects”) have also been shown to operate in some areas, whereby groups are sensitized to information that affects outcomes. The general issue is to distinguish between motivational and expectancy effects that are a natural part of an intervention (and so not to be controlled out) versus motivation bias that is the byproduct of research activities (which does need to be controlled for).

For data collected in a trial, measures may be needed to address differential motivational effects. Where it is necessary to blind, the authors should state explicitly that the process of monitoring the intervention and outcome measurement is blinded to participants, practitioners and those collecting outcomes data, and what method was used. Methods that could minimize risk of motivation bias in the absence of blinding include infrequent observation, or efforts to balance bias across groups through the same field visits and questionnaires, outcome questionnaires that avoid “anchoring bias” (e.g., avoiding leading questions that refer to the intervention), and/or use of an active control designed to generate similar motivational and expectancy effects for both groups. If the risk of bias is thought potentially severe, primary study authors may rarely adapt the study design to estimate possible motivational effects (e.g., a “pure control” with no monitoring except baseline-endline or endline only) (e.g., Banerjee et al., 2021).

Motivational biases may be less problematic when informed consent is not associated with a particular intervention (hence avoiding anchoring bias), as may be the case for a cluster-allocated trial, or a retrospectively designed evaluation using existing data (equivalent to participant blinding). Hawthorne and testing effects also tend to be short lived, because most of the behaviors that we are interested in are “sticky” (resistant to change), so they might also be addressed via longer-term (post intervention) follow-up.

#### Fidelity of the Treatment and Comparison Conditions

The key issue here is the match between the condition that was actually received by the participants and the condition that is the focus of the research question for the review. Note that this assessment is driven by the review team’s research question and not the research objectives of the primary study authors. The interest of a review team can range from the effectiveness of an intervention if delivered in ideal circumstances or the effectiveness of an intervention as it is routinely delivered in the real world (or both). Thus, things to consider are treatment fidelity and how a study handled no-shows (people who didn’t receive treatment but were assigned to treatment) and crossovers (people who received the treatment but had been assigned to the comparison condition).

These factors affect controlled studies commonly, whether they use random assignment or other assignment mechanisms. For example, systematic errors in implementation fidelity by the intervening body might affect exposure of study participants to the intervention, and therefore outcomes (Dobson & Cook, 1980). Or there might be contamination by external programs (also called “treatment confounding”), requiring an assessment of whether participants are isolated from other interventions (to avoid “substitution bias”) or exposures which might be received differentially by group. For a one-group design or time-series, the parallel issue is historical treatment confounds, such as other policies or competing interventions occurring during the study period. It is important to recognize that the causal agent being examined is the difference between the treatment and comparison conditions. Thus, the nature of both the treatment and comparison condition matter.

For the comparison condition, the key issue here is any difference between the observed comparator and the ideal comparator as articulated in the research question for the systematic review.

In many social contexts, spillover effects may occur where comparison units receive some aspect of the intervention via interactions with treatment units, or substitution bias may occur where controls obtain similar treatments from different providers (Heckman & Smith, 1995). Evaluating the fidelity of study conditions may involve assessing whether: the intervention is likely to spill over to comparison units (e.g., minimized when treatment groups are spatially separated from one another); or participants are isolated from other interventions or exposures which treatment units do not receive. Cluster-assigned treatments may be required to address these types of bias and/or an assessment of the separation of study participants. In a one-group design or time series, the parallel issue is whether changes occurred during the baseline period in anticipation of the start of the intervention or policy change.

### Adequacy of Measurement

#### Temporal Precedence

Ambiguity in whether the intervention, practice, program or policy precedes the outcome introduces a risk of bias due to reverse causation. This risk of bias is usually minimal for prospective designs but is of key concern in retrospective studies, particularly those using cross-sectional design or others that ask participants to recall information about the timing of treatment. A minimum condition for drawing causal inference is showing that the cause (intervention) came before the effect (outcome). In RCTs and most QEDs this is not an issue. However, where it cannot be shown that the treatment or policy preceded the outcome, there is a risk of bias.

#### Quality of Measurement

Measurement error and other measurement issues often receive too little attention in assessments of study validity. At the most basic level, random measurement error (random noise in a measure) moves treatment effects towards the null. This statistical phenomenon is well-established in the psychometric and econometric literatures. Almost all measures have at least some degree of measurement error and many of the outcome measures of interest in systematic reviews in the social and natural sciences have considerable error. However, measurement biases (sometimes called “systematic measurement errors”) also often exist, such as recall bias or social desirability effects. Measurement biases may increase or decrease treatment effect estimates. For such measurement biases to affect treatment effect estimates, they must act differentially between the groups (or, for one-group studies, between the pre and post measurement periods).

Poor quality measures reduce the sensitivity of a study to identifying treatment effects. Low measurement reliability and validity reduce observed effect sizes. Therefore, the key issue here is whether the measures for the outcome of interest were of high quality, that is, valid measures of the construct of interest and reliable measures or measures with little random error. Measurement scale issues to consider that may bias an effect size are range restriction, ceiling effects or floor effects. If there is a longitudinal element to the design, changes in the measurement over time are also a potential concern. For example, in an interrupted time series study, a change in how the outcome is measured may appear as a treatment effect.

Measures may also be biased when these data are obtained from the study participants (or their surrogates, such as parents, teachers or other practitioners) and when these participants are aware of their program condition. Self-reporting is hampered by expectations and social desirability effects. The key issue here is whether participants are altering their responses, even unintentionally, because they know which condition or group they are in. Note that for this to be biasing, it needs to affect the groups differently. For example, if responses are affected by social desirability but equally so in both groups, then in theory this does not bias the treatment effect estimate (although measurement error might attenuate the effect towards the null). In open (unblinded) longitudinal studies with repeated measurement, participant fatigue may cause respondents to misreport to reduce the time needed to engage further with the study (Clasen, 2013). Thus, outcomes based on self-report, other-report, or even the observations of a researcher, may be biased based on these individuals’ awareness of a participant’s condition.

As noted, the blinding of study participants to treatment condition is usually impossible and undesirable in trials in social and natural sciences and related topics. However, it may be possible to use a negative control treatment, such as math textbooks in a study of the effects of improved hygiene on diarrhea (Luby et al., 2004). In trials of community interventions (cluster assignment), it may be possible to blind those collecting data to treatment status, or not disclose the purpose of the outcomes survey in the process of obtaining informed consent from participants.

There are several other measurement issues to consider, depending on the nature and context of the research being reviewed. For example, recall bias may affect reported treatments and outcomes. Recall is hampered by knowledge of the person providing information about others and their memory of events. But it is difficult to pre-specify a recall period that is appropriate for all measurement constructs. For some measures, such as recalling household food consumption, or the symptoms of a common infection, the recall period is preferably no longer than 48 hours. For others, particularly for salient events like the death of a child, or criminal victimization, the recall period is less likely to matter (Savović et al., 2012).

A controversial measurement issue is the use of outcome measures developed by the researchers or treatment developers. Wolf and Harbatkin have shown that these measures produce larger treatment effect estimates on average (Wolf & Harbatkin, 2022). This may reflect greater sensitivity of the measure to the changes produced by the intervention (a good thing), or it may reflect a “teaching to the test” effect that does not generalize to the broader outcome of interest (a bad thing).

### Reporting of Analyses

#### Estimation Methods

A study’s statistical analysis and the reporting of that analysis can affect the credibility of the reported effect sizes. The key issue here is the use of a statistical model that is known to produce a biased estimate given the characteristics of the study and data. It is not possible to articulate all of the potential statistical issues that might be relevant for impact evaluation studies and many of the potential problems should be familiar to reviewers with a solid foundation in statistical methods. However, some issues are less obvious. For example, the treatment effect is biased if the statistical model includes a post-baseline variable (or one where the timing is ambiguous) that is caused by both the treatment and the outcome. Similarly, the effect size will be biased if it is based on a statistical model that includes treatment dosage or another mediating outcome (i.e., something caused by the treatment but not the outcome). The theory of change can be useful in evaluating these types of estimation problems.

Falsification methods, also called negative controls, can provide important supportive evidence in impact evaluations, including in RCTs where outcomes are reported (Arnold & Ercumen, 2016). For example, studies might usefully report tests for negative-control outcomes which are unrelated to the intervention according to a reasonable theory of change. The theory of change can also help in identifying likely causal pathways in mediator analysis, or in providing theoretical support for particular methods such as exogeneity of an instrumental variable.

Design-specific statistical tests should also be reported such as tests for negative control thresholds (also called “placebo discontinuities”) in fuzzy RDD. In studies using statistical matching, tests for hidden bias can be reported (Rosenbaum & Rubin, 1983). The joint significance of the instruments, goodness-of-fit of the participation equation and an over-identifying test should be reported in an instrumental variables analysis.

Many threats to equivalence of groups over time can be addressed via an intent to treat (ITT) analysis. Such an analysis compares all units (e.g., persons) assigned to treatment to all units assigned to the control, independent from whether they actually received it. Such an analysis will be unaffected by the problem of non-adherence, even in a random assignment study. Other estimates may be of interest, such as the average treatment effect on those who actually received the treatment as intended (i.e., the ATET). Simply comparing treatment completers to the comparison group may be a biased approach to such an estimate when there is non-adherence. There are statistical methods for estimating an unbiased estimate of ATET, such as the use of instrumental variables estimation, and a reviewer needs to assess the adequacy of those methods if they are used.

#### Selective Reporting

The key issue here is whether there was selective reporting of outcomes or of statistical analyses by study authors. Selective reporting of outcomes may refer to the selection of a desirable effect from among multiple possible outcomes that are collected, selective reporting of results from sub-groups of participants, or selective reporting of methods of analysis from among multiple estimation strategies or specifications (Sterne et al., 2016). The latter applies if the results are based on a complex statistical model where multiple variants of a model might plausibly have been tried. There are two main sources of this bias. The first is significance inflation (or “*p*-hacking”) whereby researchers test multiple hypotheses until they find statistically significant results, which are then submitted for publication. The second source of selective reporting is the removal of non-significant findings from the published studies, as well as the non-significant findings from studies which ultimately remain unpublished, being left in the researchers’ file-drawers (Ioannidis et al., 2017; Rothstein et al., 2005). These types of bias are particularly likely to be prevalent in retrospective evaluations, where the method of analysis or outcomes are more easily chosen based on results. But they are problematic in prospective studies as well, especially ones that are not pre-registered so there is no public record of them occurring. Presence of a study protocol (pre-analysis plan) can help determine the likelihood of bias, although it is recognized that many prospective (and nearly all retrospective) studies do not have or publish such plans, nor is it possible to fully specify all models for some methods used, especially in QEDs. When estimation does not require careful understanding of the theory of change, as in the case of RCTs, blinding of data analysts to treatment status is also feasible, although rarely done.

## Discussion

Measurement of the likely biases helps systematic reviewers assess the credibility of the inferences made in individual studies and the body of evidence as a whole. In this section we discuss practical considerations for the design and conduct of risk-of-bias assessments, and for interpreting subsequent analyses.

### Considerations for the Design and Conduct of Assessments

It should be clear that establishing whether any given design provides a credible counterfactual – that is, provides an unbiased estimate of the average potential outcome – requires that the systematic reviewer determine equivalence on more easily observable features and also evaluate the plausibility of uncontrolled sources of bias. But assessing a study’s risk of bias means evaluating the identification strategy, as well as the fidelity of the study conditions, measurement of study constructs and the reporting of analyses. The specifics of this evaluation will depend on the nature of the intervention, context, and specific design elements, including the specifics of the research question posed in the systematic review. The validity assessment will likely need to use qualitative judgment based on the theory of change, regarding which covariates should (and which should not) be incorporated in the treatment selection model, or in evaluating the conduct of particular approaches, such as that the threshold rule used in a RDD is non-manipulable by study participants or practitioners, or the likely validity of different types of self-reported outcomes. Owing to the judgment needed to assess the risk of bias, the approach should be conducted with quality controls.

We believe that the assessments should, as far as possible, avoid penalizing transparent reporting of what was done and found, nor disincentivizing future studies from doing so. In practice, this is likely to require careful judgment about what is and is not reported, including by drawing on available supplemental materials including analysis plans, with studies penalized when they do not report relevant information, where this information is not in the public domain or obtainable from authors. In other words, transparent reporting of a study becomes as important as its design and conduct. This principle is consistent with efforts being made to improve the reporting of trials and observational studies such as CONSORT and Strengthening the Reporting of Observational Studies in Epidemiology (STROBE) (Dewidar et al., 2025; Elm et al., 2007; Hopewell et al., 2025).

We suggest that users draw on the framework proposed here, further developing signaling questions that are important to the review topic. We articulate some principles we believe are important in designing and reporting appropriate assessments for use by systematic review teams:

(1) Assessments must be consistent with the types of studies that are eligible for inclusion in the review. Practically, this necessitates the inclusion of questions relevant for all eligible study designs, such as the possibilities of subverted treatment assignment in RCTs or RDDs, non-equivalent trends in pre-test outcomes in a DID study, or the use of information about the selection of units into treatment and comparison conditions when this is based on variables measured or available to the study. Assessments also need to pose appropriate questions to evaluate bias, even if some design features are impossible – for example, questions about the timing of visits to assess participant reactivity effects in trials or spatial separation in clustered treatments, rather than simple assessments of blinding.
(2) Risk-of-bias assessments should also be consistent with the guiding questions for the systematic review, so that the purpose of the systematic review is driving the decision on the importance of a design feature in assessing the risk of bias. An example is whether the purpose of the systematic review is to assess effectiveness under ideal or real-world conditions, which would affect assessments of the fidelity of treatment conditions.
(3) Consequently, it is important to avoid reporting risk-of-bias assessments as markers of the quality of a study (e.g., ‘low quality’ or ‘high quality’). The risk of bias provides the probability attached to the confidence in the study’s findings with regards to the intervention(s) and outcome(s) of interest in the systematic review (which may be different from the primary studies themselves). These ratings usually take the form of ‘low risk’, ‘moderate risk’ and ‘high risk of bias’, and some tools also incorporate ‘severe’ or ‘critical risk’ to signify those findings that are too uncertain to be included in the synthesis of findings.
(4) Finally, signaling questions should be answerable, subject to caveat, simply and consistently across coders. In practice, this can mean having multiple nested signaling questions to improve coder inter-rater reliability. Decision rules should be specified to avoid penalizing transparent reporting (and rewarding intransparency), which can necessitate deeming lack of information as equivalent to lack of compliance. For example, Cochrane’s tools use “Yes” (Y), “Probably yes” (PY), “Probably no” (PN), “No” (N), and “Not stated” (NS) or “not applicable” (NA), in the assessments.^7^ If information needed to verify the design or conduct is “not stated” in manuscript or supplemental materials (or verifiable through author correspondence), this becomes equivalent to “No” or “Probably No” in the decision rule determining the risk-of-bias rating (Sharma Waddington et al., 2025).

We do not provide in this discussion paper a tool that provides a decision algorithm linking the signaling questions to a risk-of-bias rating at the bias category and study-outcome level. It is common for reviewers (and tool designers) to apply a simple rule to determine an overall rating for each outcome reported in a study, such as the “weakest link” principle, where the overall risk-of-bias assessment can be no better than the assessment for the bias category judged the weakest.

### Interpreting Analysis of the Assessments

Empirical reviews suggest that studies with lower probability of bias are likely to estimate effects that are systematically different from studies with higher probability of bias (e.g., Sharma Waddington et al., 2022a). However, it is not clear that bias, for individual categories or overall, exerts a systematic effect on either inflating or deflating treatment effect estimates in individual studies. One might expect QEDs to have bigger effects than RCTs for two main reasons: non-adherence, which causes the intention-to-treat (ITT) estimator to be of smaller magnitude than the treatment on the treated estimate, often produced by a QED;^8^ and publication bias, which causes QED estimates to be typically larger than RCTs because the research and publication process enables RCTs to be published more easily, regardless of the study’s findings. Alternatively, “site selection bias” in trials might cause interventions to be implemented more carefully (e.g., due to a motivation effect of the study on intervention practitioners or by cultivating ideal circumstances) than routine practice, leading to bigger effects on average (Allcott, 2015; Sharma Waddington et al., 2023). Complicating matters further, in QEDs where self-selection bias leads to those with better prognostic factors being allocated to the treatment arm (and also adhering to the intervention), we would expect studies to estimate larger effects on average (Kunz & Oxman, 1998).

Thus, the relationship between the source of bias and the estimated effect is likely to vary, so that, in most cases, the answer to the question about whether bias is associated with systematic deviation in effects towards or away from the null, is that we simply don’t know. For example, if attrition or losses to follow-up were positively correlated with the outcome and greater in the treatment group (i.e., positively correlated with treatment), the effect size estimate would increase; whereas if attrition were higher in the control group (negatively correlated with treatment) then the effect for attrition positively correlated with outcome is to decrease the estimated effect. Similarly, participant motivation may increase effects (e.g., improved performance among treated units, resentful demoralization among controls, testing effects in the treatment group) or reduce them (e.g., compensatory rivalry among controls, participant fatigue due to repeated measurement, testing effects among controls).

Measurement error, however, has a known direction of bias. It is well established that random errors in measurement attenuate any observed effect towards zero, making programs appear less effective than they really are (Lipsey, 1990; Wooldridge, 2008). Similarly, spillovers (contamination of controls due to exposure to treated participants or outcomes) would tend to reduce the mean difference between treatment and control outcomes for an effective intervention, as would no-shows and crossovers (switches) when the estimated effect measured is the intention-to-treat.

The point we are making is to avoid assuming that studies at ‘high risk of bias’ will de facto over-estimate a treatment effect. A realistic assessment is that it increases uncertainty regarding the true underlying effect of an intervention, and therefore the variance. Thus, it is critical to assess sources of bias carefully, and to report the different characteristics transparently by bias category, so that decision making regarding the intervention can be appropriately situated within some bounded uncertainty.

## Conclusion

This paper discusses risk-of-bias assessment in impact evaluation in social and natural sciences, health and related fields, where a wide range of randomized and non-randomized study designs is used, and varying degrees of control for unobservable sources of bias. We present a framework for evaluating the risk of bias in these types of evaluations, which incorporates four main sources and eight categories of bias. We are careful to acknowledge that the signaling questions that are relevant for particular designs and contexts, may not be relevant for others. There may also be specific issues that are meaningful in some contexts, requiring the development of additional signaling questions, where these are seen as relevant for the review question and topic. This reinforces the idea that risk-of-bias assessment should be adapted to the specific topic and research question. It is important that impact evaluations of whatever type are reported transparently, so we have designed the questions to avoid incentivizing weak reporting in future studies. We suggest authors of experiments, quasi-experiments, natural experiments and observational studies of interventions in social and natural sciences and related fields consider the sources of bias presented here when designing, conducting and reporting on their studies.

## Supplemental Material

Supplemental Material - Risk of Bias in Experiments, Quasi-Experiments and Natural Experiments Across Disciplines: Discussion Paper and Assessment Framework Supplemental Material for Risk of Bias in Experiments, Quasi-Experiments and Natural Experiments Across Disciplines: Discussion Paper and Assessment Framework by Hugh Sharma Waddington, David B. Wilson, Terri Pigott, Ariel M. Aloe, Peter Tugwell, Vivian Welch, Gavin Stewart in Campbell Systematic Reviews.

## Notes

1. Natural experiments are defined as studies where the researcher does not play a role in manipulating exposure to treatment (Craig et al., 2025). They are usually designed retrospectively utilising exposure and outcomes data available from routine sources.

2. This assessment drew on the bias categories identified in commonly used risk-of-bias tools including (Cochrane Effective Practice and Organisation of Care (EPOC), 2017; Hombrados & Waddington, 2012; Sterne et al., 2019).

3. Randomization does not, however, guarantee balance of all factors in any single draw, hence RCTs often stratify on observables, or less commonly conduct multiple draws.

4. Regardless of the type of treatment assignment mechanism used, where participation is voluntary there is still likely to be self-selection of participants into or out of treatment or adherence during the study, including when assignment is targeted.

5. See also (Özler, 2012).

6. A negative control outcome is often called a “placebo outcome”. We avoid using this term as we believe it reflects a mischaracterization of placebo effects, which are real effects on the outcome of interest.

7. This is the approach used in ROBINS-I and ROB (Sterne et al., 2016, 2019).

8. In practice, the distinction is fuzzy. For example, RCTs with non-adherence may be analyzed using instrumental variables, or, incorrectly, in ATET. Similarly, the ITT can be estimated in some QEDs. In the case of RDD, the estimate may be of a local average treatment effect (LATE) applicable to a particular group around the treatment threshold, which may not be applicable to the whole study population average treatment effect (ATE).
