# Supplementary material for: Risk of Bias in Experiments, Quasi-Experiments and Natural Experiments Across Disciplines: Discussion Paper and Assessment Framework
Source: Campbell Syst Rev. 2026 Apr 18;22(2):18911803261435894. doi: 10.1177/18911803261435894 (PMC13170436; doi:10.1177/18911803261435894)
Supplement: Supplemental Material - Risk of Bias in Experiments, Quasi-Experiments and Natural Experiments Across Disciplines: Discussion Paper and Assessment Framework [file sj-pdf-1-cam-10.1177_18911803261435894.pdf]

Table A1 Bias categories and signaling questions by source of bias

Bias source	*Signaling question*	*Explanation*	*Applicable designs*
**1. Equivalence of groups**	**Baseline equivalence**		
	Was the integrity of the random allocation (or other allocation method) to conditions maintained?	The integrity of an RCT is directly related to the integrity of the initial random assignment to conditions. Deviations from the random allocation protocol can introduce bias (non-random differences between the groups). Assessing this requires that the authors describe how they randomized study units into conditions, whether there were departures from this process, and how they were handled. For prospective (non-fuzzy) RDDs, the integrity of the design is directly related to adherence to the assignment threshold rule on a scale variable at baseline.	This only applies to RCTs and prospective RDDs.
	Does the study incorporate all known or suspected sources of difference between groups that are also potentially related to the outcome of interest?	A major concern in quasi-experimental comparison-group designs is whether the design or analysis accounts for unobserved baseline variables that are related to both whether units (people, areas, communities, organizations, etc.) are part of the treatment or comparison condition and the outcome of interest. To be problematic, an unobserved variable must be related to both the selection mechanism into or out of treatment and the outcome variable of interest. Information about the process of allocating treatment units, such as the program targeting mechanisms used by planners (e.g., using a proxy means pre-test measure or geographical targeting), can usefully be incorporated into the statistical model estimating the treatment effect, thereby reducing bias. For analysis using instrumental variables or another "exogenous" identifier, it is important qualitatively to establish that the identifier is external – that is, it is only causally related to the outcome by determining selection	Designs with a contemporaneous comparison group that do not use either random assignment to conditions or threshold assignment.

Bias source	*Signaling question*	*Explanation*	*Applicable designs*
		to treatment.	
	Are the groups similar to each other on observed baseline characteristics?	The core issue is the comparability of the groups at baseline in a comparison group design. The focus is on the size of any observed difference and not on whether these differences are statistically significant. The latter is highly dependent on sample sizes, thus potentially penalizing large sample size studies relative to small sample size studies. Even in an RCT, there will be baseline differences. The critical question is whether these differences are large enough to potentially bias the results. A meaningfully large difference on an observed variable may also signal a difference on one or more unobserved variables. For educational interventions, the U.S. What Works Clearinghouse standard for baseline equivalence is a difference of 0.25 standard deviations or less. Additionally, this standard requires that treatment effect estimates are statistically adjusted for any difference between 0.05 and 0.25 on a baseline variable.	Designs with a contemporaneous control or comparison group.
	Are there sufficient pre-test measurement time points to establish a credible baseline counterfactual?	For these designs, the baseline period functions as the comparison or counterfactual. The assumption is that without the introduction of the intervention, the underlying trend would have remained the same. Thus, among other things, the number of pre-test measurement points affects the ability to predict what would have happened without the intervention effectively. The number of pre-test measurement time points needed depends on the specific design and intervention context. For example, a study in health systems research found that at least six observations, both before and after the policy was implemented, were needed to generate unbiased effects in ITS analysis.(Fretheim et al., 2015)	One-group designs with historical comparisons: one-group, pre-post designs, interrupted time series designs, and single-subject designs.

Bias source	*Signaling question*	*Explanation*	*Applicable designs*
	**Selection into and out of the study**		
	Were there potential participants (or other study units) for whom the outcome could not be assessed or who joined after the study began?	Ideally, the estimate of the treatment effect will be based on an analysis that includes the outcome for all study units present at condition allocation (i.e., baseline). Differential selection into the study by groups – e.g., due to "survivor bias" – may occur before baseline (e.g., deaths or migration of eligible participants) or after it (e.g., where individual participants are selected after treatment assignment). A related issue, including in cluster randomized trials, is joiners. For example, many educational experiments randomize classrooms or schools to intervention and control groups. Students joining classrooms after the start of a study (i.e., after random assignment) could compromise the integrity of random allocation depending on the nature of the treatment (What Works Clearinghouse, 2022). This applies to cluster assigned interventions on many other social policy and related topics.	Designs with a contemporaneous comparison group and cluster-assigned trials.
	Were there study participants (or other study units) who were lost to follow-up (attrition)?	Selection out of the study is referred to as losses to follow-up or attrition bias. The loss of study participants (or other study units) can erode the comparability of the groups, introducing bias. The key issue is whether any loss of study units from either condition and for any reason is potentially different between conditions and of a sufficient magnitude to bias the estimate of the treatment effect, but overall attrition if large may also be problematic. Differential attrition in excess of 10 percentage points and/or overall attrition in excess of 20 percent of observations, are thought potentially problematic, particularly where information is not provided on the causes of losses to follow-up by group or tests are not done of the relationship between attrition and baseline measures. Missing data may also relate	Designs with a contemporaneous control or comparison group.

Bias source	*Signaling question*	*Explanation*	*Applicable designs*
		to the complexity of the measure taken.	
**2. Fidelity of study conditions**	**Participant reactivity**		
	Is it plausible that participants' reactions to being part of a research study affects their behavior?	When participants know they are part of a study, it may affect how they behave, particularly if they know what treatment condition they belong to. This category of biases includes compensatory rivalry or demoralization on the part of the control group, and improved performance among the treatment group simply because they know they are being studied. The key issue here is the potential reactivity of study units to being part of a research study, a reactivity that might be heightened where there is repeated and regular observation. Opening the intervention black box to articulate the nature and frequency of observation can help in assessing this question. Some studies use "active controls" who receive the same number of visits by programs and research staff to foster balance in motivation effects due to repeated observation. These effects also tend to be short lived, so measurement post-intervention (i.e., longer term follow-up) can help allay concerns about them.	Prospective designs with a contemporaneous control or comparison group.
	In a one-group design or time series, are there concerns that changes occurred in anticipation of the start of the intervention or policy?	In one-group designs, where policy changes are anticipated, perhaps in response to a recent increase or decrease in some outcome of public interest, this introduces the possibility of an anticipated effect on outcomes during the pre-treatment period. As such, it is important to assess the plausibility that the timing of the outcome was affected by knowledge of a forthcoming policy.	This applies to one-group historical comparison designs.
	**Treatment and comparison fidelity**		
	Are there concerns	This question is about the match between the treatment condition as	This applies to all

Bias source	*Signaling question*	*Explanation*	*Applicable designs*
	regarding the match between the treatment that is the focus of the research question for the systematic review, and the treatment that was actually received by the participants?	delivered in the study and the treatment construct of interest. It is important to note that the focus is on how well the treatment as delivered matches the research question for the review. Thus, this assessment is driven by the review team's research question and not the research objectives of the primary study authors. The interest of review teams may range from the effectiveness of an intervention delivered with high fidelity or they may be interested in the effectiveness of an intervention as routinely delivered in the field. The key issue here is the fit between the treatment of interest to the research question for the review and the treatment as delivered in the study. It is also possible that a study reports high fidelity but for a treatment program that differs from the canonical program of interest to the review, articulated in the theory of change.	study designs.
	Are there concerns regarding the match between the comparator condition actually received, and the comparator condition as articulated in the research question for the systematic review?	In a comparison group design, the effect being estimated is defined by the contrast between the groups. Thus, the nature of both the treatment condition and the comparison condition matters. Comparison conditions may be active, such as treatment as usual or an alternative treatment, or inactive (not treatment). Comparison conditions that do not match well the contrast of interest can bias the effect estimate. The focus is on how well the comparator condition as implemented in the study matches the canonical comparator condition of interest to the review.	This applies to all contemporaneous control or comparison group designs.
	Is there a risk that the treatment spills over to the control or comparison condition?	In some contexts, participants receiving the treatment may provide information about the intervention to comparison group participants. The key issue is whether one person or study unit’s receipt of the treatment affects the outcome of any other study unit. For example, in a school context, students might discuss what they learned in an intervention program with students not in the program. In economics	This applies to all contemporaneous control or comparison group designs.

Bias source	*Signaling question*	*Explanation*	*Applicable designs*
		and environment, the concern is often with spatial closeness or the adequacy of spatial separation.	
	Were there things that happened differentially to the groups other than the treatment versus comparison distinction?	The key issue here is whether there is anything that is confounded with the treatment/comparison contrast that might affect outcomes. This may affect observations at the individual or group levels. For example, factors affecting outcomes may occur differentially across clusters of observations, such as weather events in studies of agriculture or the environment.	This applies to any contemporaneous comparison group design.
	Are there other policies or competing interventions occurring during the study period?	A potential risk of bias in one-group type designs is whether some other competing treatment, social policy or event is confounded with treatment. This creates ambiguity as to what produced any change that occurred over time. That is, was it the intervention of interest or the confounding treatment? Assessing this based on a written report for a single-group design can be difficult as it often requires knowledge of what else might have been co-occurring with the intervention within a specific study context (and this information is often not provided in a theory of change). Treatment confounding is less problematic in comparison group studies but may still occur in designs of complex interventions where the intervention of interest, such as a group-based batterer intervention program, is confounded with other differences between the groups, such as a court mandate.	This applies especially to one-group designs, but may in practice apply to all quasi-experiments.
**3. Adequacy of measurement**	**Temporal precedence**		
	Is there a clear temporal ordering of the treatment and the outcome?	The issue is that, to identify a treatment's effect, causes must precede effects. Thus, any ambiguity in the temporal precedence of the treatment or policy relative to the outcome introduces a risk of bias.	This applies to retrospectively designed studies,

Bias source	*Signaling question*	*Explanation*	*Applicable designs*
		For RCT designs and many prospective quasi-experimental designs, the intervention clearly comes before the outcome. However, temporal precedence may not be clearly established for retrospective studies and designs based on cross-sectional data.	and may be particularly relevant to designs using cross-section data.
	In a one-group design or time series, is it implausible that the intervention or social policy was introduced in response to a recent increase or decrease in the outcome of interest?	In one-group designs, interventions may follow a recent increase or decrease in some outcome of public interest, such as a sudden increase in street crime. This introduces the possibility of a regression-to-the-mean artifact. As such, it is important to assess the plausibility that the timing of the intervention was a reaction to recent changes in the outcome of interest.	This applies to one-group historical comparison designs.
	**Quality of measurement**		
	Do the measures of treatment have poor accuracy?	The key issue is whether information on the treatment condition assigned or received is accurate, especially where information on dose, frequency, intensity or timing are needed. Measures with low measurement reliability are "noisy" measures, that is, they have a lot of random error in them. As such, they underestimate treatment effects relative to a more reliable measure of the same construct. A key source of bias here is recall bias. This is less problematic where information is collected at the time of the intervention from sources not affected by outcomes (e.g., enumerators). It is potentially problematic where information about treatment status is obtained after implementation from participants or practitioners who may misremember in recall or have an incentive to misreport.	This applies to all designs.
	Do the outcome measures have poor reliability?	The key issue is whether information on the outcome measures is reliable. Measures with low measurement reliability are noisy. As	This applies to all designs.

Bias source	*Signaling question*	*Explanation*	*Applicable designs*
		such, they under-estimate treatment effects relative to a more reliable measure of the same construct.	
	Do the outcome measures have poor validity?	Measures with low validity may not capture the change of interest, thus, underestimating the treatment effect. A key source of bias here is recall bias. A key source of bias here is recall bias. This is potentially problematic where information about outcomes is obtained from participants or practitioners who may misremember in recall. This is less problematic where the outcome is salient and measured as the incidence of an event (e.g., violent crime, mortality).	This applies to all designs.
	Does the nature of the outcome measure(s) change over time?	The key issue is whether what appears to change over time might be the result of changes in how the outcome is measured. This is particularly an issue with designs relying on administrative data where how information is coded into the information system changes over time. An alternative form of this is whether the intervention changes peoples' understanding of outcome questions, thus introducing a potential source of bias.	This applies to all designs with multiple measurement time points, whether prospective or retrospective.
	Does the person providing the outcome data know which condition the participant or unit was in?	The key issue is whether knowledge of which condition a participant or unit is in might subtly affect the assessment of the outcome. This can be controlled through blinding of the assessor (masking to condition). If the data are based on self-report, is it plausible that an individual's awareness of the program that they received influenced their responses? Are results from negative controls (non-equivalent outcome functions) presented?	This applies to all designs.
	Is the outcome measure over-aligned with the treatment?	The key issue is whether each condition had equal exposure to the content of the outcome measure. For example, in an educational intervention, the outcome measure may test content provided to the	This applies to all designs.

Bias source	*Signaling question*	*Explanation*	*Applicable designs*
		treatment condition, but not to the control condition.	
	Is the outcome measure developed by the researchers and/or developers of the intervention?	The key issue is whether the outcome represents a broader set of knowledge and skills as represented by a standardized measure with associated reliability and validity evidence. When the outcome measure was developed by the treatment developers or by the researchers, the outcome may be biased in favor of the treatment group.	This applies to all designs.
**4. Reporting of analyses**	**Estimation methods**		
	Does the statistical model that is estimating the treatment effect exclude any post-baseline variable that is part of the causal pathway between the treatment and the outcome?	Including post-baseline variables in a statistical model can bias the treatment effect estimate. An example is including treatment dosage in the same model that is estimating the overall treatment effect. Similarly, the treatment effect is biased if the statistical model includes a post-baseline variable (or one where the timing is ambiguous) that is caused by both the treatment and the outcome.	This applies to all designs, but may be particularly relevant for contemporaneous control or comparison group design.
	Are the statistical tests appropriate to the study design reported?	Design-specific statistical tests include negative control thresholds (also called “placebo discontinuities”) in fuzzy RDD; tests for hidden bias in studies using statistical matching; tests for joint significance of the instruments, goodness-of-fit of the participation equation and an over-identifying test in instrumental variables analysis.	This applies to all designs, but may be particularly relevant for contemporaneous control or comparison group design.
	Does the study provide	The key issue here is whether the effect size that is computed from a	This applies to all

Bias source	*Signaling question*	*Explanation*	*Applicable designs*
	sufficient statistical information to compute an effect size adjusted for observed baseline data?	quasi-experimental comparison group design reflects the full strength of the design. For example, does the computed effect size take into account baseline covariates used by the study to estimate the treatment effect or is it based simply on the raw mean difference? A strong quasi-experimental design might provide an effect size that is at high risk-of-bias if it does not reflect the study's most credible estimation method.	quasi-experimental designs with contemporaneous comparison groups.
	Is the change in outcome likely to occur over the study period, rather than reacting slowly or continue to change after treatment?	The key issue is whether delays in effects due to slow implementation or adherence, would mask the treatment effect, particularly where it is measured at a single point in time. As such, it will be important to model the evolution of lagged outcomes appropriately. Understanding the intervention theory of change might help assess this.	This particularly applies to one-group historical comparison group designs, but in practice may apply to all designs.
	Does the estimation of the treatment effect rely on an intent-to-treat analysis, rather than the average treatment on the treated?	It is common for study participants to not receive the intended treatment. Dropping these cases from the estimation of the treatment effect can introduce bias by producing groups that are no longer equivalent. In other words, participants who decide to adhere to a treatment program, switch treatment arms or stop participating in it, often differ from those who complete a program. As such, the estimation of the treatment effect should be based on an intent-to-treat (ITT) analysis that includes all participants present at baseline and assumes they received the intended treatment.	Designs with a contemporaneous control or comparison group.
	**Selective reporting**		
	Is there selective reporting of statistical analysis?	The key issue here is whether there was selective reporting of statistical analyses by study authors. The latter applies if the results	This applies to all designs.

Bias source	*Signaling question*	*Explanation*	*Applicable designs*
		are based on a complex statistical model where multiple variants of a model might plausibly have been tried. Selective reporting of statistical analyses is a form of publication selection bias. If only a subset of analyses is reported, it is likely that these are the analyses that showed the largest effects. This may only affect post-hoc subgroup analyses that are not the focus on the systematic review. However, if the main results of interest to the systematic review are based on a selectively reported analysis, then there is risk-of-bias. Were governance processes in place to manage how analyses and findings are reported (such as a protocol or pre-analysis plan)?	
	Is there selective reporting of outcome measures?	The key issue here is whether there was selective reporting of outcomes. The latter applies in studies where multiple outcomes are collected and tests can be done on each. Were deviations from the study protocol or pre-analysis plan adequately explained? Was there blinding of data analysts (masking to treatment conditions)?	This applies to all designs.
